# Anticancer evaluation of new organometallic ruthenium(ii) flavone complexes†

**DOI:** 10.1039/d2md00304j

**Published:** 2022-12-15

**Authors:** Mai Khater, John A. Brazier, Francesca Greco, Helen M. I. Osborn

**Affiliations:** a School of Pharmacy, University of Reading Whiteknights Reading RG6 6AD UK f.greco@reading.ac.uk h.m.i.osborn@reading.ac.uk; b Therapeutic Chemistry Department, Pharmaceutical & Drug Industries Research Division, National Research Centre Cairo Egypt

## Abstract

Targeting multiple malignancy features such as angiogenesis, proliferation and metastasis with one molecule is an effective strategy in developing potent anticancer agents. Ruthenium metal complexation to bioactive scaffolds is reported to enhance their biological activities. Herein, we evaluate the impact of Ru chelation on the pharmacological activities of two bioactive flavones (1 and 2) as anticancer candidates. The novel Ru complexes (1Ru and 2Ru) caused a loss of their parent molecules' antiangiogenic activities in an endothelial cell tube formation assay. 1Ru enhanced the antiproliferative and antimigratory activities of its 4-oxoflavone 1 on MCF-7 breast cancer cells (IC_50_ = 66.15 ± 5 μM and 50% migration inhibition, *p* < 0.01 at 1 μM). 2Ru diminished 4-thioflavone's (2) cytotoxic activity on MCF-7 and MDA-MB-231 yet significantly enhanced 2's migration inhibition (*p* < 0.05) particularly on the MDA-MB-231 cell line. The test derivatives also showed non-intercalative interaction with VEGF and c-myc i-motif DNA sequences.

## Abstract 2

Targeting multiple malignancy features such as angiogenesis, proliferation and metastasis with one molecule is an effective strategy in developing potent anticancer agents.

## Graphical abstract

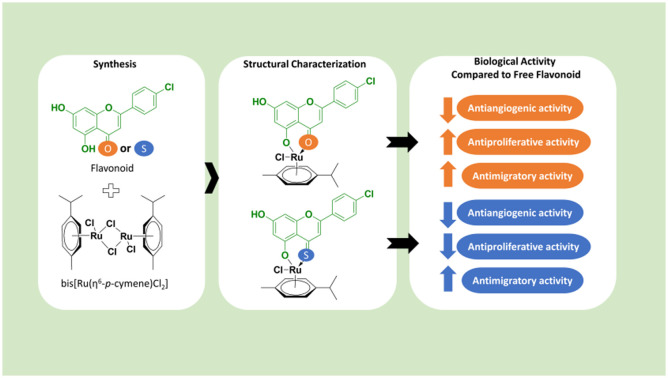

## 1. Introduction

**Fig. 1:**
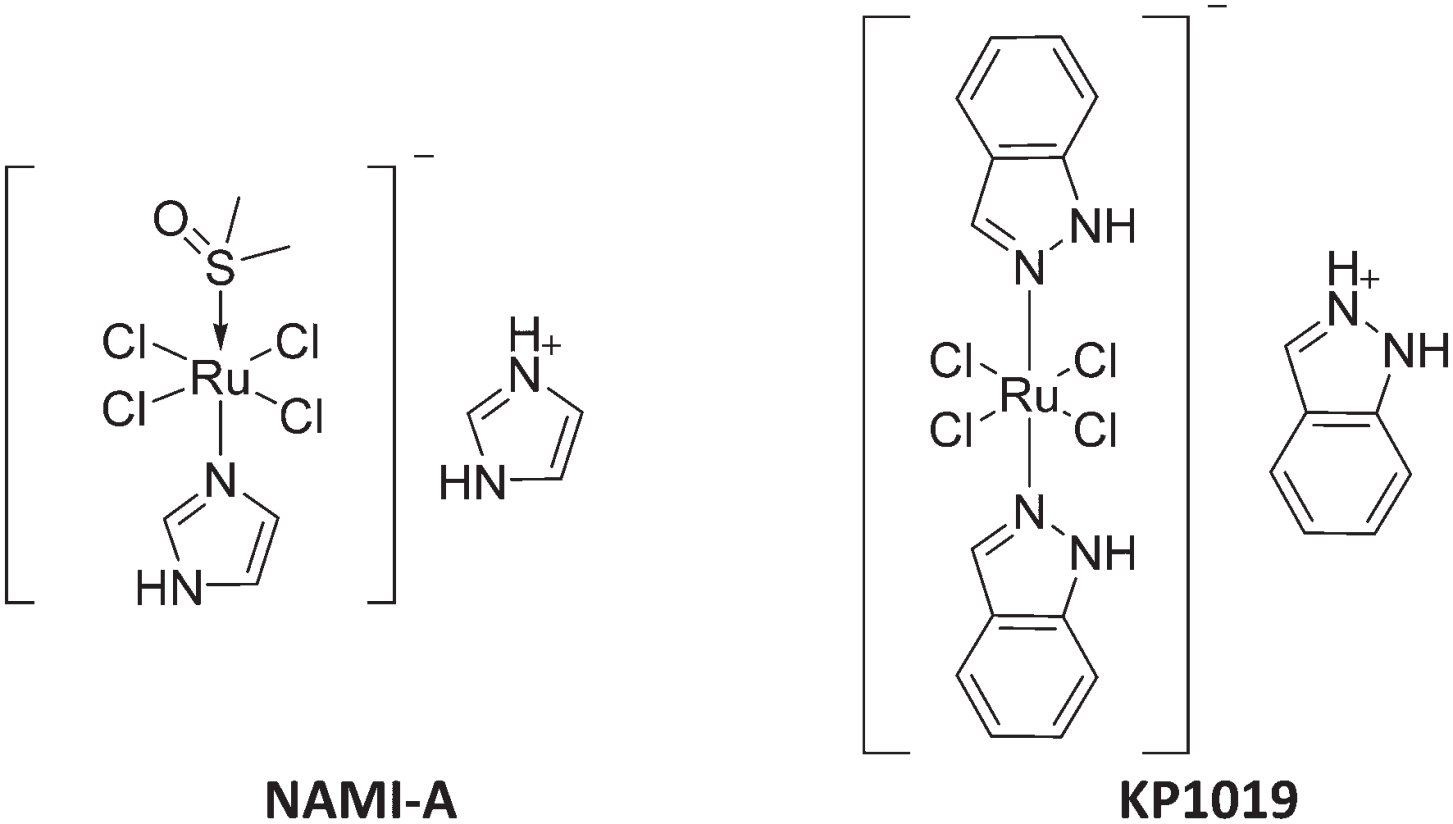
Chemical structures of NAMI-A and KP1019.

In spite of the current advances in cancer chemotherapy, treatment of metastatic tumours remains challenging. In that context, 90% of cancer related mortalities are caused by metastatic tumours rather than the primary malignant tissue.^1^ Tumours rely on the constant formation of new blood vessels in order to grow and metastasize.^2^ A proangiogenic switch plays a critical role in the growth of malignant tissues by increasing their supply of oxygen and nutrients. Additionally, increased angiogenesis promotes the invasive properties of dormant cancer cells which contributes to their metastatic profiles.^3^ Since the discovery of the DNA binding agent cisplatin, the search for novel metal-based anticancer agents has evolved towards the development of less toxic versions of metal-based anticancer drugs with high efficacy and lower susceptibility to resistance.^4^ Ruthenium based compounds in particular have gained special interest due to their desirable pharmacological properties such as high potency, low toxicity and low predisposition to resistance.^5,6^ The Ru(iii) organometallic compounds NAMI-A^7^ and KP1019^8^ were the first Ru metal-based derivatives to reach cancer clinical trials and display strong *in vivo* antimetastatic and anticancer activities, respectively^9^ (Fig. 1). Despite the preliminary encouraging results, both NAMI-A and KP1019 did not proceed further with clinical trials due to toxicity and solubility issues.^5^

In depth research on the mechanisms of action of Ru(iii) metal complexes showed that they are activated *in vivo via* reduction of the inert Ru(iii) to the more toxic state, Ru(ii).^10,11^ Owing to their lower oxidation states, Ru(ii) derivatives are more thermodynamically and kinetically stable than Ru(iii) complexes.^12,13^ Consequently, the attention of researchers has shifted towards the direct use of the active Ru(ii) organometallic complexes especially in the form of arene-Ru(ii) complexes. The unique half sandwich stool geometry and [η^6^-arene-Ru(ii)-XYZ] formula of arene-Ru complexes provides valuable structural features for medicinal chemistry programmes. For example, the arene ring, which comprises the seat part of the stool conformation, can increase cellular uptake of the metal complex by increasing its lipophilicity. X and Y are either one bidentate or two monodentate ligands and together with Z (a leaving group, such as a halogen) they constitute the legs of the stool conformation.^14–16^ Structural tuning of any part of the pharmacophore (*i.e.* the arene ring, the ligand and the leaving group) can alter the biological activity of the entire complex and its mode of action. *O*,*O*-Chelated-ruthenium complexes are widely reported for their cytotoxic and antimetastatic activities.^14,17,18^ Pettinari *et al.* reported *in vitro* cytotoxic effects of biphenyl pyrazolonate based, and curcumin based, *O*,*O*-chelated ruthenium complexes in two studies.^19,20^ The pyrazolonate derivatives showed activities comparable to that of cisplatin on cervical, breast, hepatocellular and colorectal cancer cell lines (IC_50_ values of 9–34 μM compared to 13–52 μM for cisplatin)^19^ while the curcumin based complexes exhibited higher potency and cancer cell selectivity than cisplatin on ovarian cancer cell lines (IC_50_ values of 0.14–1.18 μM compared to 1.5–25 μM for cisplatin).^20^

Natural products have made many valuable contributions in the field of cancer drug discovery. For example, plant based drugs such as vincristine, paclitaxel and irinotecan have formed an integral part of cancer treatment regimens.^21^ Flavonoids are polyphenolic chromone based compounds of natural origin and are capable of coordinating with metal atoms *via* their OH and/or C=O groups. Given the well reported anticancer and antiangiogenic properties of flavonoids,^22,23^ their *O*,*O*-chelation with the bioactive Ru(ii)-*p*-cymene moiety in a hybrid organometallic molecule may result in synergistic effects and enhance the pharmacological activities of both scaffolds.^24,25^ In fact, Ru metal chelation to flavonoids has been shown to enhance some of their therapeutic effects in several studies.^24,26^ Kurzwernhart *et al.*, for instance, reported better cytotoxic activity of a series of *p*-cymene-Ru(ii)-flavonol complexes than their unsubstituted flavonols on a range of cancer cell lines.^27^ However, in some cases no added benefits were observed in terms of activity after complexation hence a further study is warranted in this area to fully probe this phenomenon.

The study reported herein examined the effect of *p*-cymene-Ru(ii) metal complexation on the *in vitro* antiangiogenic, antiproliferative, antimetastatic and DNA binding activities of the flavones (1 and 2). Compound 2 is a 4-thioflavone that showed significant *in vitro* antiproliferative, antiangiogenic and VEGFR2 phosphorylation inhibition activities in our previous studies.^23,28–30^ Furthermore, the 4-oxoflavone (1) was included in the study to gain additional insight on the contribution of the functional group at position number 4 not only on the free flavonoid's activity but also on the biological effects of complexation. Initially, a tube formation assay was utilised for the synthetic derivatives (1 and 2) alongside the novel complexes (1Ru and 2Ru) to establish their antiangiogenic potential on endothelial cells which are major contributors to the spread and growth of tumours. The direct effects of the synthesized compounds on malignant cells' proliferation were evaluated *via* cytotoxicity studies on the estrogen receptor positive (MCF-7) and the triple negative (MDA-MB-231) breast cancer cell lines. Due to the key role of cell migration in cancer metastasis, antimigratory activities of the flavone parents and the corresponding Ru(ii) complexes were assessed on both the MCF-7 cell line and the invasive MDA-MB-231 cell line. Structure activity relationships (SAR) and dose response associations were also examined to gain insight on the antimigratory behaviour of these ruthenium metal derivatives and their parent flavones. Binding interactions of the synthetic flavone derivatives with the telomere i-motif forming sequences of VEGF and c-myc oncogenes (overexpressed in many cancers) were studied *via* UV-visible and DNA melting spectroscopy. I-motif DNA sequences are presumably capable of regulating oncogene expression and therefore are of interest for their anticancer applications.^31,32^

## 2. Results and discussion

### 2.1. Synthesis

**Scheme 1:**
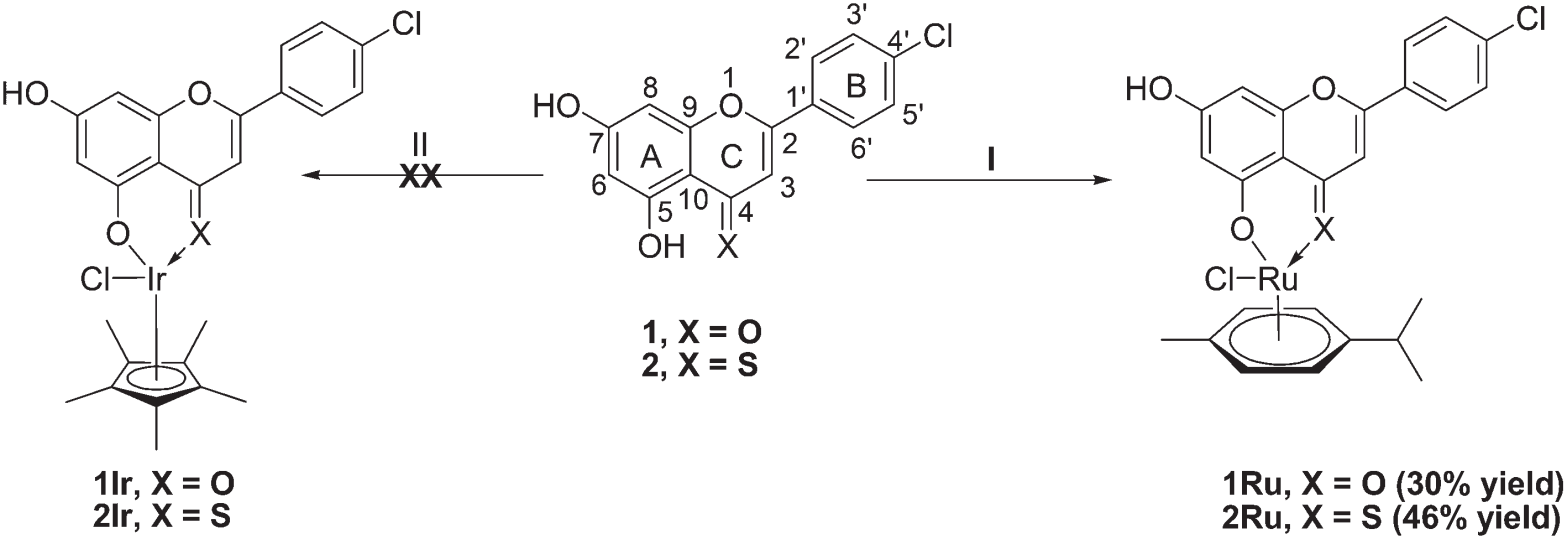
Synthesis of Ru(ii) flavone and thioflavone derivatives. (I) NaOMe, [Ru(η^6^-*p*-cymene)Cl_2_]_2_ in anhydrous DCM, MeOH, 75 °C, 18 h; (II) NaOMe, [Ir(η^5^-Cp*)Cl_2_]_2_ in anhydrous DCM, MeOH, 75 °C, 18 h.

The novel Ru (η^6^-*p*-cymene) flavone complexes 1Ru and 2Ru were synthesized *via* the reaction of their parent flavones 1 and 2, respectively, with the commercially available bis[Ru(η^6^-*p*-cymene)Cl_2_] following the deprotonation of 1 and 2 by sodium methoxide (NaOMe) in methanol (Scheme 1) based on the reported method.^16^ The 4-C=O and 4-C=S ruthenium derivatives (1Ru and 2Ru) were purified by crystallization in 9 : 1 ethyl acetate (EtOAc) : acetonitrile (ACN) or EtOAc : chloroform (CHCl_3_), respectively, in 30% and 46% yields. Structures of the synthesized organometallic complexes 1Ru and 2Ru were confirmed by ^1^H and ^13^C NMR spectroscopic analysis, infrared spectroscopy, mass spectrometry, and elemental analysis. The ^1^H NMR spectrum of 1Ru showed the appearance of the *p*-cymene CH_3_ protons peaks at *δ* 1.29, 1.30 and 2.17 ppm in addition to a multiplet corresponding to the CH proton at 2.84 ppm. Furthermore, aromatic protons of the cymene ring appeared as two doublets at *δ* 5.37 and 5.66 ppm (*J* = 7.2 Hz). Successful chelation of 1 and Ru(ii)-*p*-cymene was also reinforced by the upfield shift of all of the proton signals of compound 1 with the ring A protons (H-6 and 8) seeing the highest shifts by 0.3 and 0.45 ppm, respectively. The peak of the 5-OH group proton where the chelation occurred was no longer present in 1Ru's ^1^H NMR spectrum. Likewise, the ^1^H NMR spectrum of 2Ru exhibited the same patterns of successful complexation where signals of the *p*-cymene protons appeared at *δ* 1.16 and 1.18 (2xCH_3_), 2.26 (CH_3_), 2.83 (CH), 7.07 and 7.11 (Ar–CH) ppm in addition to the thioflavone moiety protons that were slightly shifted upfield and the disappearance of the 5-OH proton (ESI† Fig. S1B).

Fig. S3 and Table S1 in the ESI† document show the UV-vis stability profiles of the synthesized complexes 1Ru and 2Ru where they've shown minor spectral changes upon temperature increments indicating sufficient stability.

The carbon signals for the *p*-cymene ring were visible in both the aliphatic and aromatic regions along with the *p*-chlorophenyl ring carbons of the flavone moiety in the ^13^C NMR spectra of both complexes. For complex 1Ru for instance, the ^13^C NMR spectrum showed peaks corresponding to the CH_3_ and CH groups of cymene at *δ* 17.92, 22.52 and 30.89 ppm in addition to the aromatic cymene carbons at *δ* 102.70, 106.34 and 129.81 ppm whereas a downfield shift of the flavone peaks was observed especially with ring A and C carbons (C8, 6, 3 and 5). The 4-C=O group signal also shifted from *δ* 181.83 to 177.55 ppm in the ^13^C NMR spectrum of 1Ru.

Attempts to synthesize the corresponding iridium(iii) derivatives of flavones 1 and 2 did not afford the desired products. Following the same synthetic route detailed for the successful synthesis of the ruthenium(ii) complexes, 1 and 2 were activated by deprotonation with NaOMe and this was followed by the addition of bis[Ir(η^5^-Cp*)Cl_2_] *in situ*. Interestingly, the ^1^H NMR spectrum of 1Ir showed 2 signals for the 5xCH_3_ protons of the Cp* ring at *δ* 1.55 and 1.64 ppm and those corresponding to the flavone scaffold as well (ESI† Fig. S2A). This finding was previously reported for the related Ir(iii)chrysin^33^ and indicates the afforded product is a mixture of 1Ir complex and starting material, or a mixture of the bidentate and monodentate Ir(iii)-flavone complexes making its purification attempts unsuccessful. Although ^1^H NMR spectroscopic analysis of the 4-thioflavone complex (2Ir) demonstrated successful chelation with a singlet signal for the 5xCH_3_ protons of the Cp* ring at *δ* 1.64 ppm, in addition to the expected flavone moiety proton peaks (ESI† Fig. S2B), its CHN elemental analysis mainly corresponded to the bisdichlorido(η^5^-Cp*)iridium(iii) starting material indicating failed complexation.

### 2.2. Cytotoxicity on HUVEC cells

**Fig. 2:**
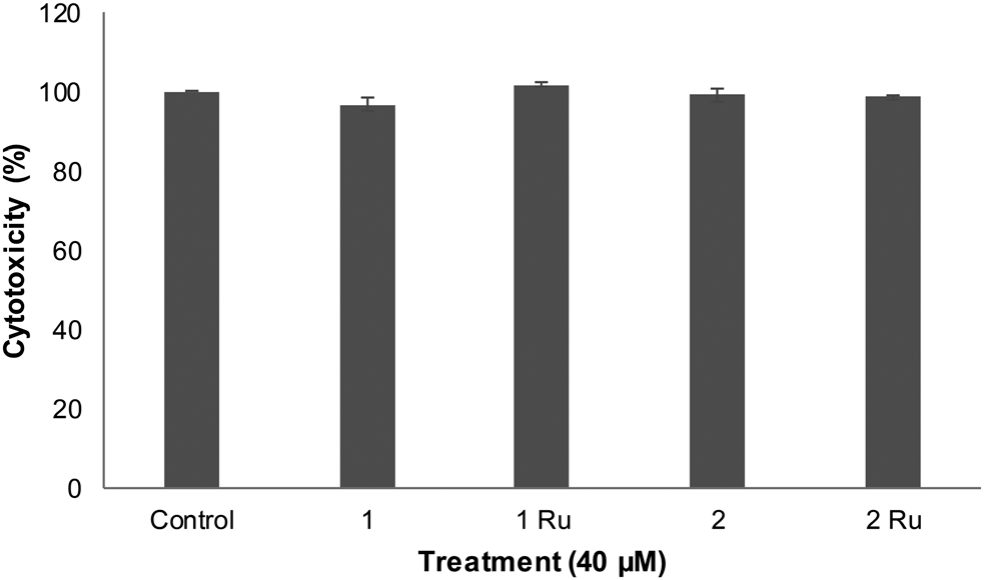
Cell viability of HUVECs at 40 μM of tested flavones. Data are expressed as mean ± standard error of the mean (SEM), *n* = 3.

The biocompatibility of flavones (1 and 2)^30^ alongside their ruthenium(ii) metal complexes (1Ru and 2Ru) with HUVEC cells was established using the trypan blue exclusion assay. A concentration four times higher (*i.e.* 40 μM) than the highest concentration used for the tube formation antiangiogenic evaluation study (*i.e.* 10 μM) was selected for this cytotoxic assay. Fig. 2 shows that the tested compounds retained ∼100% viability of the cells with no statistically significant differences from the control (*p* > 0.05) indicating suitability of the test concentration (10 μM) for the antiangiogenic evaluation using HUVEC cells.

### 2.3. *In vitro* tube formation assay

**Fig. 3:**
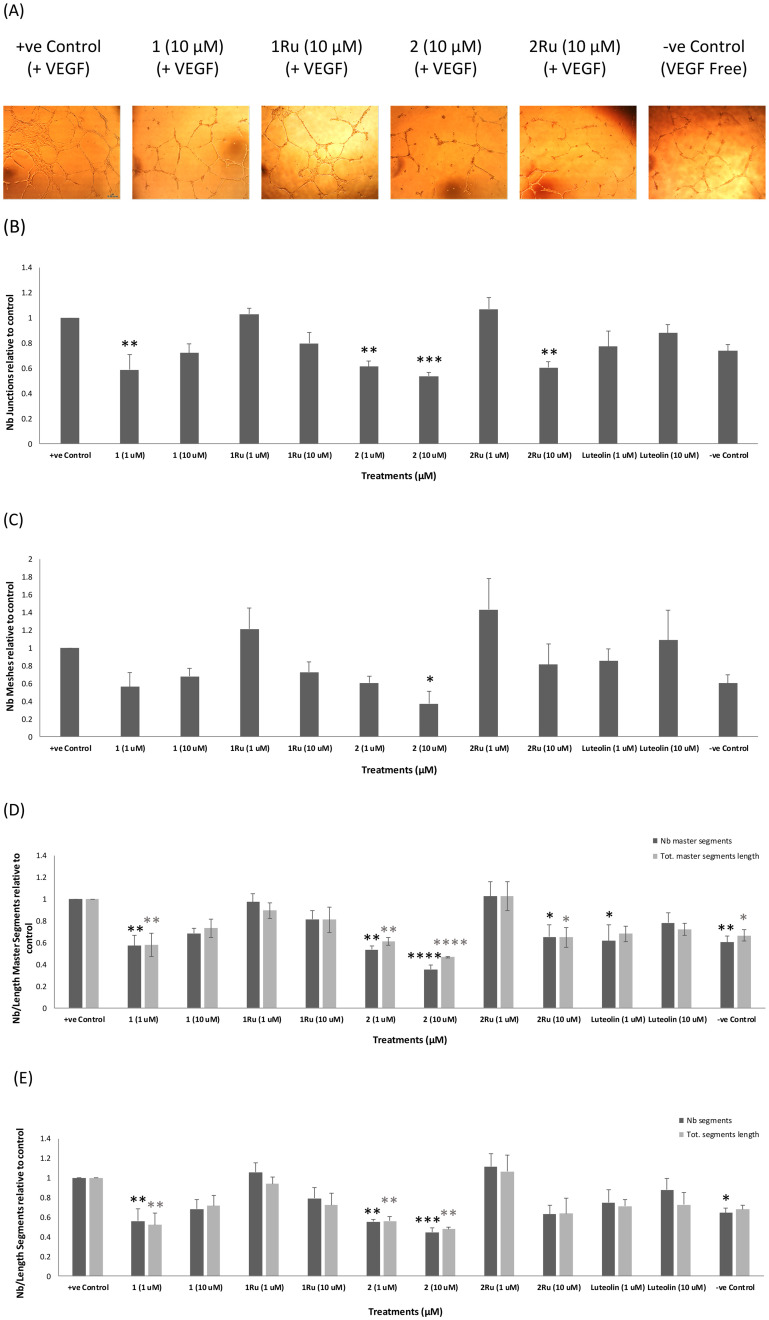
Antiangiogenic activity of Ru(ii) complexes (1Ru and 2Ru) compared to the reported activity of their parent flavones (1 and 2) on *in vitro* HUVEC tube formation after 12 h expressed as a ratio to the +ve control (10 ng mL^−1^ VEGF-enriched media). (A) Representative images of tube formation assay at 4× magnification. Images were analysed using angiogenesis analyzer macro in ImageJ software; (B) number of junctions, (C) number of meshes; (D) number and length of master segments, (E) number and length of segments. Data are expressed as mean ± standard error of the mean (SEM), *n* = 3. Statistical significance was estimated with respect to the +ve control by one-way ANOVA, followed by Dunnett's multiple comparison test (*p < 0.05, **p < 0.01, ***p < 0.001, ****p < 0.0001).

In our recent study,^30^ the 4′-chlorophenylflavone derivatives 1 and 2 have shown promising antiangiogenic activities *in vitro*. Herein, the effect of complexation with a ruthenium(ii) metal ligand on the antiangiogenic activity of 1 and 2 was evaluated using the Matrigel tube formation assay that assesses many of the main steps involved in the process of angiogenesis. Inhibition of VEGF-mediated tube formation by the tested substrates was observed after 12 h of treatment on HUVECs at 1 μM and 10 μM concentrations. Luteolin is a natural flavone well known for its high antiangiogenic activity both *in vitro* and *in vivo*^34^ and as such was used in this study as a reference standard. In accordance with reported data, luteolin inhibited the measured elements of tube formation by 30% compared to the positive control.^34,35^ As seen in Fig. 3, the 4-thio-flavone-ruthenium complex 2Ru, exhibited significant reduction in the number of junctions (40%, *p* < 0.01) and both the number and length of master segments (35%, *p* < 0.05) at 10 μM. As for the carbonyl complex (1Ru), even though it showed some antiangiogenic activity at 10 μM (overall tube formation inhibition = 22%), the observed effects were insignificant (*p* > 0.05) across all the measured elements of tube formation. Meanwhile, both complexes 1Ru and 2Ru showed a total loss of their parents' tube formation inhibition activity at 1 μM (Fig. 3). In our latest study, compound 2 was shown to mediate its antiangiogenic effects mainly *via* interaction with the VEGF/VEGFR2 pathway where the 4-C=S had a key role in the interaction with VEGFR2 and subsequent inhibition of its phosphorylation.^30^ In this regard, complexation of a Ru(ii) ligand at the 4-C=S group of compound 2 might have masked its desirable effect and lead to an altered binding mode with VEGFR2 which resulted in the significant reduction in activity seen upon complexation. The ability of Ru(ii) organometallic derivatives to target tumour metastasis has been widely reported both *in vitro* and *in vivo*.^3,6,9,36^ Thus, based on the preliminary antiangiogenic trends observed for our target Ru(ii) complexes (1Ru and 2Ru) and their parent flavones (1 and 2), we further investigated their antiproliferative and antimetastatic potential on the estrogen receptor positive breast cancer cell line (MCF-7) and the more aggressive triple negative cell line (MDA-MB-231).

### 2.4. Cell viability study

The unique chemical features and coordination geometries of metal ions can add several additional binding interactions that can potentially enhance their anticancer properties for example by allowing intercalation and exploitation of their redox potential.^24^ Lead compound (2) has previously exhibited promising anticancer activity on several cancer cell lines in the low μM range.^29^ In that context, coordination of derivative (2) with a Ru(ii) metal ligand is a promising approach to further improve its antiproliferative activity. Likewise, structural modification of the inactive flavone (1) *via* ruthenium complexation can presumably result in a new lead with higher activity. Hence, the cytotoxic activities and IC_50_ values of complexes (1Ru and 2Ru) and flavones (1 and 2) were measured by a 72 h MTT assay using a maximum concentration of 100 μM.

**Fig. 4:**
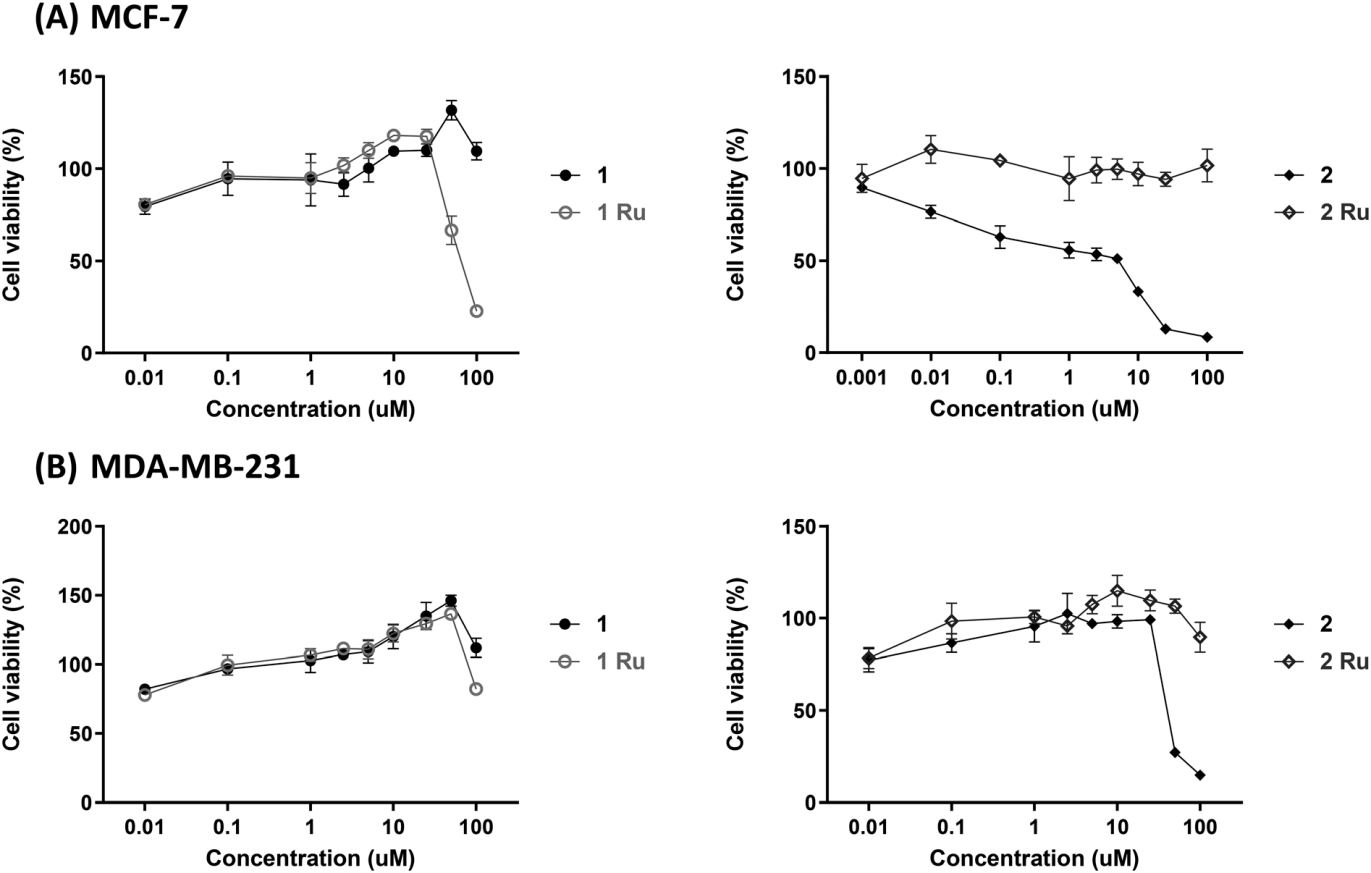
Antiproliferative activity of flavone derivatives (1 and 2) and their Ru(ii) complexes (1Ru and 2Ru) against (A) MCF-7 and (B) MDA-MB-231 cancer cell lines. Data are expressed as mean ± standard error of the mean (SEM), *n* = 3.

As shown in Fig. 4A, free flavone (1) showed no cytotoxic activity above 100 μM on the two breast cancer cell lines whereas its Ru(ii) complex (1Ru) showed enhanced antiproliferative activity but only on the estrogen receptor positive cell line (MCF-7) with an IC_50_ of 66.15 ± 5 μM. In contrast, complex 2Ru diminished the high antiproliferative activity observed for derivative 2 on the MCF-7 breast cancer cell line (IC_50_ = 1.2 ± 0.8 μM) and the moderate activity on the triple negative MDA-MB-231 cell line (IC_50_ = 43.06 ± 1.29 μM) (Fig. 4B, Table 1). Since substitution of the 4-C=O with 4-C=S group is the only structural difference between compound 1 and 2, the S atom is likely to be critical for compound 2's cytotoxic activity. This observation explains, in part, the loss of the cytotoxic activity that occurred after complexation with Ru in complex 2Ru as complexation might have hindered the S atom's ability to interact with its cellular target(s). The negative impact that masking of the 4-C=S group had was also demonstrated earlier when probing the antiangiogenic activity of 2Ru*via* the tube formation assay. Moreover, several ruthenium organometallic complexes, such as NAMI-A, have similarly shown low potency in *in vitro* cytotoxicity studies while exhibiting remarkable antimetastatic effects *in vivo*.^14,37^ Hence, the antimetastatic potential of complexes 1Ru and 2Ru in addition to their free flavones 1 and 2, was evaluated by measuring their effects on breast cancer cells' migration.

**Table 1:** Determined IC_50_ for flavone derivatives (1 and 2) and their Ru(ii) complexes (1Ru and 2Ru) against MCF-7 and MDA-MB-231. Data are expressed as mean ± standard error of the mean (SEM), *n* = 3

Compound	IC_50_ (μM)	
	MCF-7	MDA-MB-231
1	>100	>100
1Ru	66.15 ± 5	>100
2	1.2 ± 0.8	43.06 ± 1.29
2Ru	>100	>100

### 2.5. Wound healing (migration) assay

**Fig. 5:**
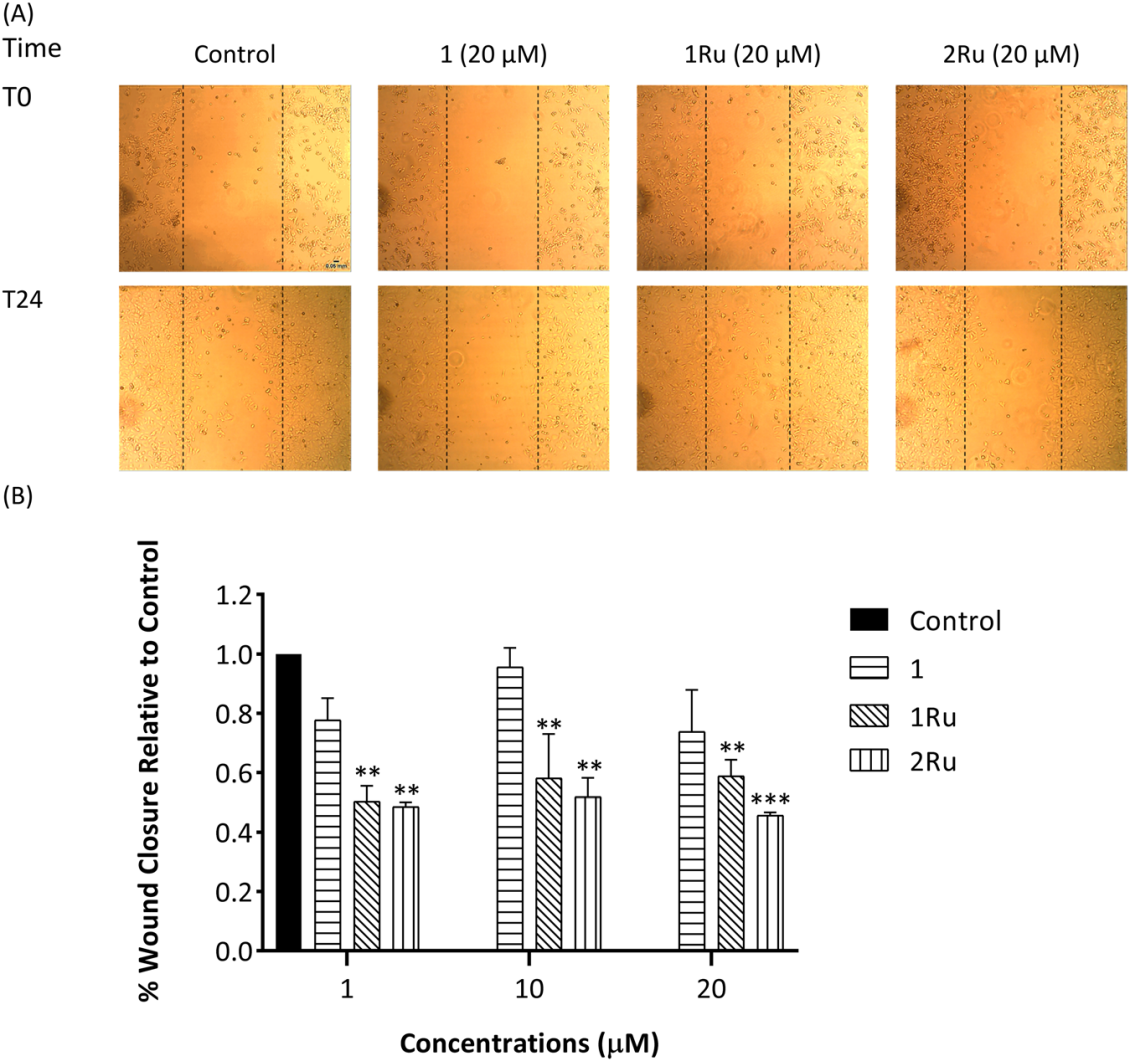
*In vitro* MCF-7 wound closure (migration) inhibition activity of flavone derivative (1) and the Ru(ii) complexes (1Ru and 2Ru) expressed as a ratio to control. (A) Representative images of scratch assay at 0 h and 24 h at 4× magnification. Images were analysed using ImageJ software; (B) % wound closure after 24 h as a ratio to the control. Data are expressed as mean ± standard error of the mean (SEM), *n* = 3. Statistical significance was estimated with respect to the +ve control by one-way ANOVA, followed by Dunnett's multiple comparison test (**p < 0.01, ***p < 0.001).

Malignant cell migration is essential for tumour invasion and metastasis.^1^ Thus, the ability of the synthetic flavones (1 and 2) and their Ru(ii) metal complexes (1Ru and 2Ru) to inhibit the migration of breast cancer cells (MCF-7 and MDA-MB-231) was assessed by an *in vitro* 24 h scratch assay. Low, sub-cytotoxic concentrations of 1, 10 and 20 μM were used in this evaluation. Given the high toxicity of compound 2 on the MCF-7 cell line (IC_50_ = 1.2 ± 0.8 μM), it was not possible to measure its antimigratory effects on that cell line. The 4-thioflavone ruthenium complex (2Ru), showed 55% migration inhibition of MCF-7 cells at 20 μM (*p* < 0.001) which is the highest activity observed on this cancer cell line (Fig. 5). 2Ru's high activity continued to be significant even at the lower concentrations of 1 and 10 μM (52% and 49% inhibition, respectively, *p* < 0.01). As seen in Fig. 5B, the improvement of the antimigratory activity of compound 1 upon complexation was prominent as there were no observed effects for the parent flavone at any of the tested doses. In that regard, complex 1Ru exhibited significant reduction (*p* < 0.01) in MCF-7 cells' migration at all the tested concentrations with 50%, 42% and 41% inhibition at 1, 10 and 20 μM, respectively.

**Fig. 6:**
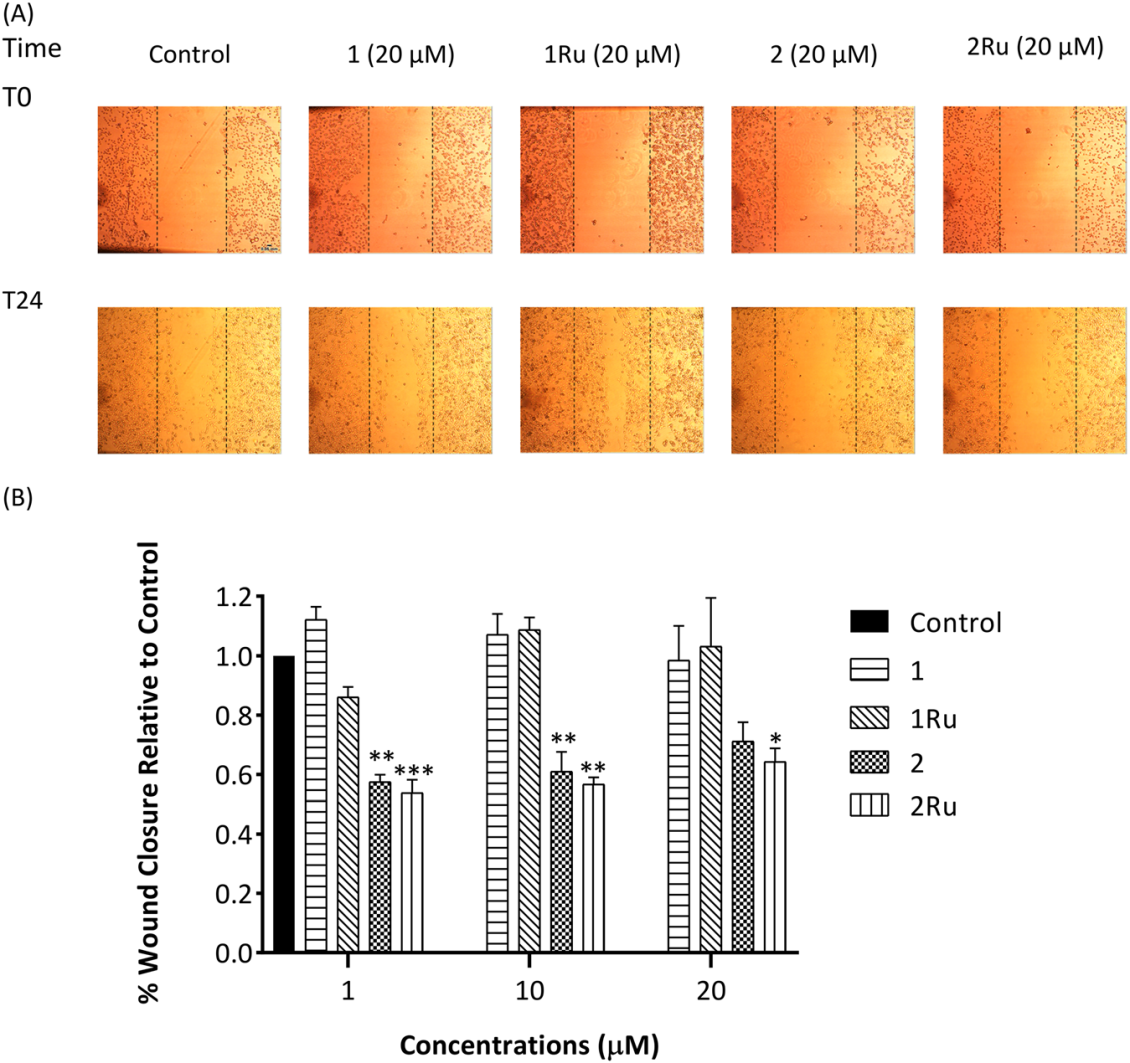
*In vitro* MDA-MB-231 wound closure (migration) inhibition activity of flavone derivatives (1 and 2) and their Ru(ii) complexes (1Ru and 2Ru) expressed as a ratio to control. (A) Representative images of scratch assay at 0 h and 24 h at 4× magnification. Images were analysed using ImageJ software; (B) % wound closure after 24 h as a ratio to the control. Data are expressed as mean ± standard error of the mean (SEM), *n* = 3. Statistical significance was estimated with respect to the +ve control by one-way ANOVA, followed by Dunnett's multiple comparison test (*p < 0.05, **p < 0.01, ***p < 0.001).

Results of the wound healing assay on the invasive breast cancer cell line (MDA-MB-231) showcased the antimigratory potency of complex 2Ru. This is highlighted by a 47% fall in MDA-MB-231's migration (*p* < 0.001) relative to the control at low levels of 1 μM (Fig. 6). 2Ru sustained its high activity at the higher 10 and 20 μM concentrations although to a lower extent (migration inhibition = 44%, *p* < 0.01 and 36%, *p* < 0.05, respectively). In terms of the biological effect of metal coordination, it resulted in a statistically significant rise in compound 2's activity of 4% and 7% (*p* < 0.05) at 1 and 20 μM, respectively. For the 4-carbonyl derivative (1) and its Ru(ii) complex (1Ru), neither showed any effect on the migration of MDA-MB-231 breast cancer cells indicating 1Ru's selectivity on MCF-7 cancer cell line. From a structure–activity relationship perspective, these findings suggest that the 4-thio group and the Ru(ii) *p*-cymene ligands improve the antimigration activity of this panel of flavones and their structural features are therefore favourable over their 4-carbonyl or un-complexed flavones. Replacement of the 4-C=O with a 4-C=S functionality has been widely reported to enhance many of the pharmacological activities attributed to flavones such as neuroprotective, anticancer and antimicrobial activities.^29,38^ The cymene-Ru(ii) moiety also add several benefits to the chelated ligand as mentioned earlier. Thus combination of the S atom with the Ru(ii)-arene presumably leads to better interaction with target proteins *via* several ways such as increasing the lipophilicity of the ligand.^30,39^

**Fig. 7:**
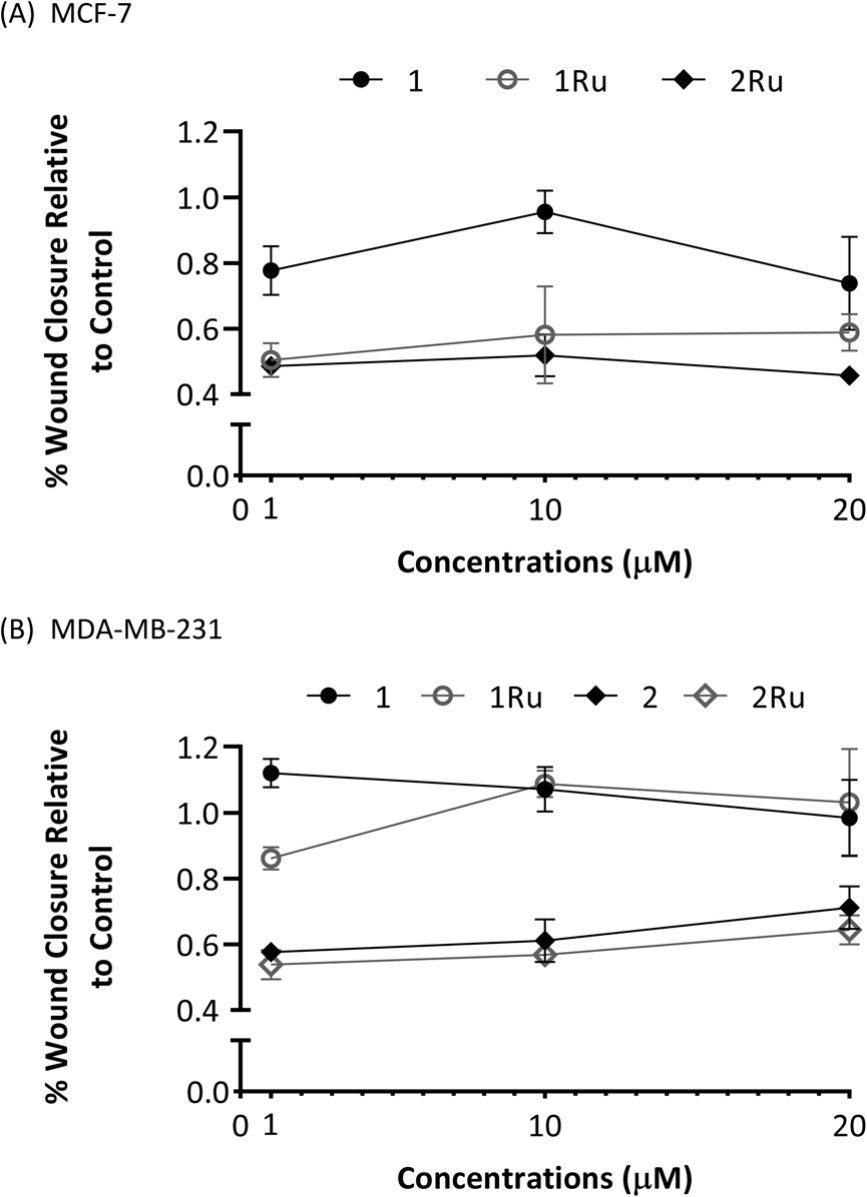
Dose response trend lines of flavone derivative (1) and the Ru(ii) complexes (1Ru and 2Ru) at 1, 10 and 20 μM concentration. (A) MCF-7; (B) MDA-MB-231.

The observed trends in antimigratory activities on both breast cancer cell lines were not concentration dependant. As shown in Fig. 7, the effects the tested compounds had on cell migration were largely comparable across the range of the different concentrations used. Data from the wound healing assay on the MCF-7 cell line (Fig. 7A) suggested that activities were at their highest levels at 1 μM, slightly decreased at the 10 μM then increased at 20 μM. With the exception of flavone 1, the highest activities on the inhibition of MDA-MB-231 cells' migration occurred at the lowest dose of 1 μM (Fig. 7B) where it was significantly higher (*p* < 0.05) in case of both 2 and 2Ru compared to their activities at 20 μM. Previous studies have reported a negative dose–response relationship of flavonoids or their metabolites on their cell adhesion and antiangiogenic effects.^30,40^ However, further investigations are still needed to unfathom the algorithms of this dosage-activity interplay.

### 2.6. I-motif DNA binding study

**Fig. 8:**
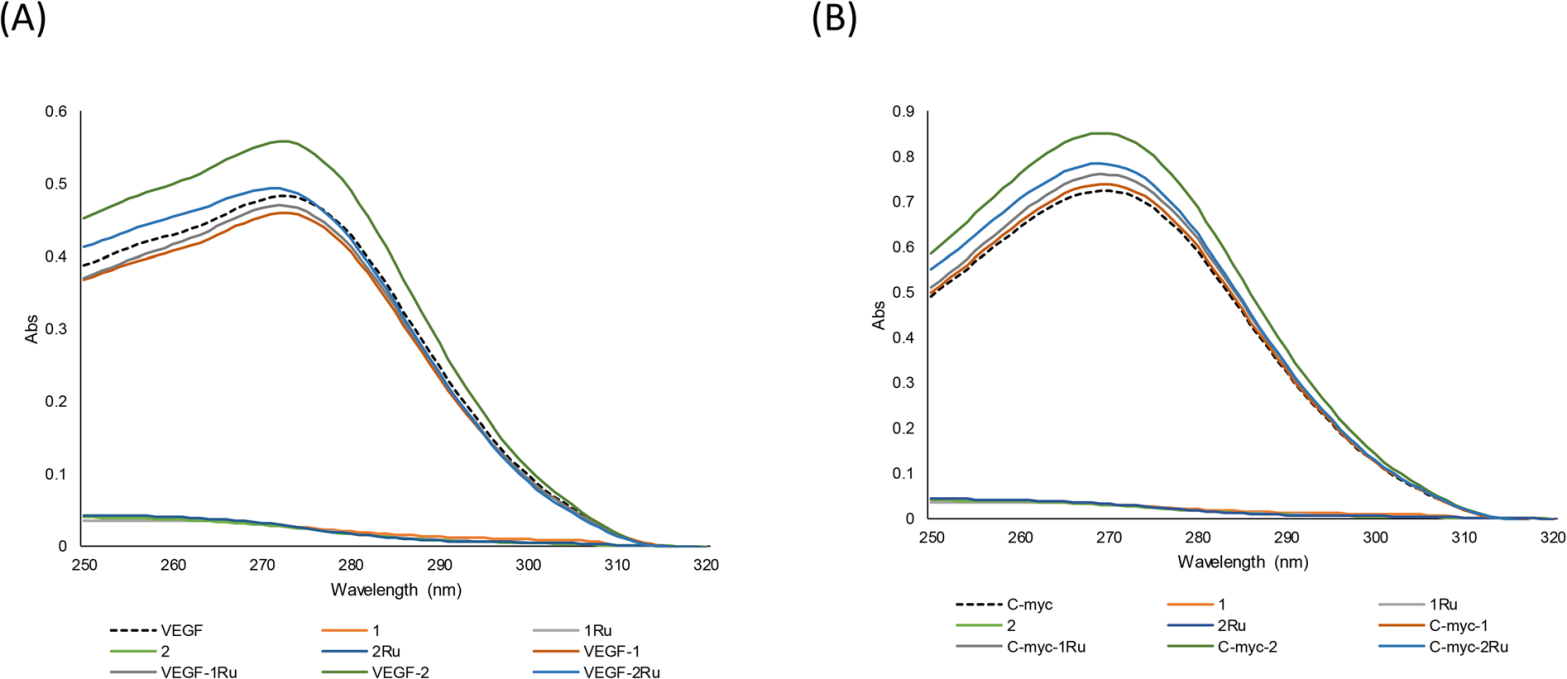
Absorption spectra of VEGF and c-myc I motif DNA with flavones (1, 1Ru, 2 and 2Ru) in 1 : 1 ratio. (A) VEGF i-motif interactions; (B) C-myc i-motif interactions.

I-motifs are four stranded antiparallel cytosine rich DNA sequences capable of forming at telomere and promotor regions of genomic DNA.^41,42^ I-motifs were initially thought to form in only acidic pH conditions, however they have recently been shown to form at physiological pH.^43^ Due to the presence of potential i-motif forming sequences in 69% of promoter regions in human oncogenes, they are postulated to play a key role in oncogene expression regulation.^44^ VEGF and c-myc i-motif sequences are prevalent in oncogene promotor regions in many cancers including breast cancer and as such their ligands have potential anticancer roles.^45^ The natural flavonol, fisetin, is reported to specifically bind to VEGF i-motif DNA and regulate its function by stabilizing its hairpin structure.^31^ Similarly, Ru-based complexes exhibited i-motif binding properties.^46,47^ In continuation of our investigation of the effects of Ru metal complexation on the different biological activities of flavonoids, interactions with VEGF and c-myc i-motif DNA were studied using UV-vis spectroscopic techniques. Fig. 8 shows the absorption spectra of VEGF and c-myc i-motif sequences with or without flavones (1 and 2) and their Ru complexes (1Ru and 2Ru) in DNA : flavonoid equimolar ratios (2 μM : 2 μM). The thioflavone (2) exhibited the most pronounced effects on the absorbance maximum (*λ*_max_) of VEGF and c-myc i-motifs with 16% and 18% hyperchromic shifts, respectively. Ruthenium metal complexation to compound 2 had a negative influence on its i-motif DNA interaction where 2Ru showed 2% and 8% increase in *λ*_max_ of VEGF and c-myc sequences, respectively. However, 2Ru's addition led to a 1 nm hypsochromic shift of VEGF i-motif absorbance maximum from 272 to 271 nm. On the other hand, addition of the 4-oxo flavone (1) and its Ru complex (1Ru) led to minor changes in *λ*_max_ of both sequences that was hypochromic (5% and 3%) with VEGF and hyperchromic (2% and 5%) in case of c-myc (Fig. 8).

**Fig. 9:**
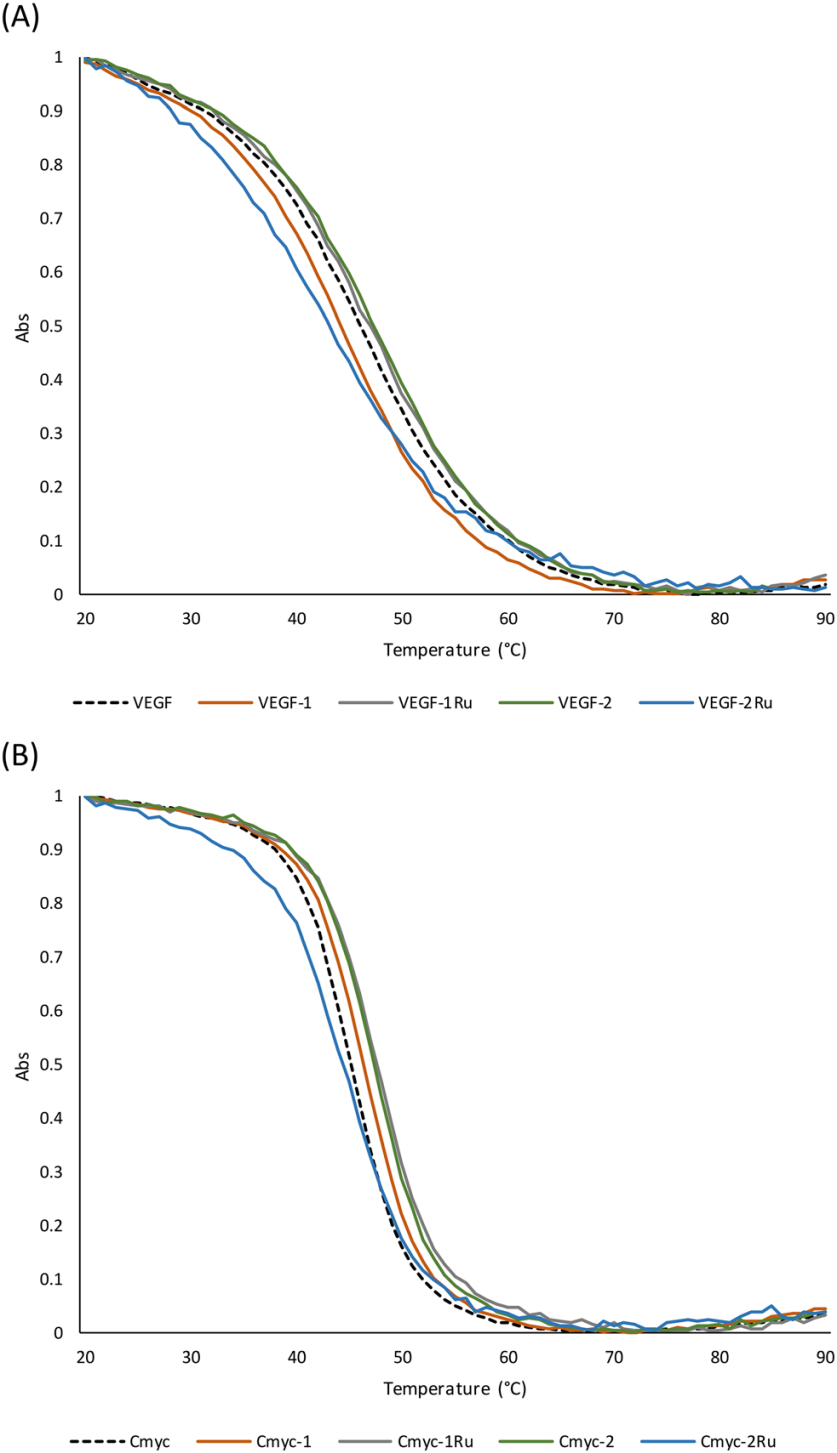
Normalized UV melting curves for VEGF and c-myc i-motif DNA with flavones (1, 1Ru, 2 and 2Ru) at 295 nm at 1 : 1 ratio. (A) VEGF i-motif melt; (B) C-myc i-motif melt.

The effect of this set of flavones on VEGF and c-myc was further evaluated *via* thermal stability measurements. Stabilizing/destabilizing the promotor i-motif structures is often reported to regulate expression of the corresponding genes, which for VEGF and c-myc could have detrimental effects on cancer growth and propagation.^48^ As observed in Fig. 9, effects of the test flavones on the thermal stability of the VEGF and c-myc i-motif sequences were generally marginal and in agreement with *λ*_max_ chromophoric shifts. Complex 2Ru, resulted in the highest effect seen on VEGF i-motif by destabilizing the sequence as indicated by 4 °C decrease in the i-motif's transition temperature (*T*_m_) (Table 2). A similar destabilization was observed for the same complex (2Ru) on c-myc however much less pronounced. Compound 1 destabilized VEGF i-motif in contrast to stabilizing the c-myc one. This mirrored the trend of a higher chromophoric shift observed for flavone 1 with VEGF i-motif sequence that also differed in pattern (hypochromic) from the shift witnessed with the c-myc sequence (hyperchromic). These findings could indicate higher selectivity of the 4-oxo derivative (1) towards VEGF i-motif based on an intercalative binding mode especially that hypochromic shifts are reported with DNA intercalation.^49,50^ In contrast to its parent, complex 1Ru showed a smaller stabilization effect (1 °C) on VEGF compared to c-myc i-motif (3 °C) again echoing the *λ*_max_ shifts. In this regard, the highest i-motif activity seen for the parent flavonoids was switched from VEGF to c-myc with compound 1 and *vice versa* with compound 2 upon Ru chelation. This emphasizes the key role of the interplay between the substitution at position 4 and metal complexation on the interaction with biological targets.

**Table 2:** *T*_m_ a and Δ*T*_m_^b^ of VEGF and c-myc i-motifs in the presence of flavones (1, 1Ru, 2 and 2Ru) at 1 : 1 ratio

VEGF i-motif	*T*_m_ (°C)	Δ*T*_m_ (°C)	C-myc i-motif	*T*_m_ (°C)	Δ*T*_m_ (°C)
VEGF	46	0	Cmyc	45	0
VEGF-1	44	−2	Cmyc-1	46	1
VEGF-1Ru	47	1	Cmyc-1Ru	48	3
VEGF-2	47	1	Cmyc-2	47	2
VEGF-2Ru	42	−4	Cmyc-2Ru	44	−1

a *T*_m_ is the midpoint of the transition from each melting experiment.

b Δ*T*_m_ = *T*_m (DNA-flavone)_ − *T*_m (DNA)_.

Previous studies have reported small changes of the *T*_m_ value of i-motif sequences with terbium (Tb) and Ru complexes in spite of good binding affinity to the target i-motif DNA. Two amino acid-Tb complexes were shown to bind to i-motif at 22 and 30 μM while having Δ*T*_m_ of −0.5 and −4 °C, respectively.^51^ A polypyridyl-Ru complex also showed high affinity to i-motif DNA at 5.6 μM while showing no effect on the thermal stability of the i-motif.^47^ Hence, despite the minor effects observed for the test flavones and their Ru complexes on i-motifs' absorbance and thermal stability, they might be indicative of some level of DNA interaction. A small increase or decrease, similar to that observed with this panel of flavone derivatives, in absorbance at *λ*_max_ is often attributed to a non-intercalative or external binding mode to DNA that is weaker in comparison to intercalation.^52,53^ Such external DNA binding has previously been reported for Ru complexes since the presence of a metal atom facilitates external DNA binding by electrostatic interaction with the negatively charged phosphate groups of DNA base pairs.^24,46,47,52^ Moreover, only a limited number of i-motif ligands have been identified throughout the literature which is often attributed to the difficulty of stacking of planar molecules inside the i-motif's compact structure.^32^ This further supports the hypothesis that the tested panel of flavone derivatives mainly display non-intercalative binding modes.

## 3. Conclusion

The influence of Ru metal complexation on the biological activities of parent flavonoids is somewhat controversial. While some reports show enhanced activities, others indicate no significant benefits upon complexation. In this study, two novel Ru(ii)-flavone complexes (1Ru and 2Ru) were synthesized and different aspects of their anticancer effects (antiangiogenic, antiproliferative, antimetastatic and i-motif binding) were assessed in comparison to their parent flavones (1 and 2). In general, complexation had a detrimental effect on the endothelial cell antiangiogenic activities of the free flavones. Further investigations of the impact of Ru metal coordination on the cytotoxic and antimigratory activities of the flavones of focus on breast cancer cell lines (MCF-7 and MDA-MB-231) displayed interesting trends. The impact of Ru coordination on the antimetastatic capacity of compound 1 mirrored the effects seen on the antiproliferative activity, with 1Ru displaying significantly enhanced activities for flavone 1 (*p* < 0.01), and improved selectively on the MCF-7 cell line. On the other hand, 2Ru reduced the antiproliferative activity of its unchelated flavone (2) yet showed high inhibition of both breast cancer cells' migration which was significantly better than its parent 2 on the MDA-MB-231 cell line (*p* < 0.05). Interactions of flavones (1 and 2) and their Ru complexes (1Ru and 2Ru) were also studied with VEGF and c-myc i-motif DNA using UV-vis spectroscopic techniques. The tested derivatives and their metal complexes have shown minor shifts in the absorption maxima and the transition temperatures of the i-motif sequences mostly indicating a non-intercalative mode of binding. These initial findings on the UV-vis effects of these flavones on VEGF and c-myc i-motif DNA sequences need further exploration in scope of the mode of interaction as well as the concentration and pH dependency of these effects. The patterns of activity resulting from the different assays used in this study highlight the important role of the 4-functional group on the antiangiogenic, cytotoxic, antimetastatic and DNA binding activities of the tested panel of derivatives. First of all, a positive role of the 4-C=S group was observed on the overall activity of this set of flavones with an additional benefit of Ru(ii) complexation on the antimigratory activity. Secondly, the similarity between the cytotoxic and migration inhibition trends for 1Ru suggest a common antiproliferative/antimetastatic target that is selective for MCF-7 cells. This is indeed opposed by the variability in these trends witnessed with the thiocarbonyl counterparts (2 and 2Ru) hence, proposing different antiproliferative/antimetastatic mechanisms of action that might be shared between both breast cancer cell lines. All in all, these results emphasized the constructive contribution of metal complexation on flavones' antimetastatic activities, and the fundamental role of the ligand's chelating atom as well as the nature of the studied pharmacological effect on the biological outcome. Hence, this study paves the way for the development of novel bi-modal anticancer/antimetastatic agents^54^ that are able to combat both tumour growth and propagation. Such leads with dual activity are much needed in the fight against cancer since they allow for the use of fewer chemotherapeutic agents and consequently reduce the undesirable side effects and clinical implications of using combinations of different drugs. Moreover, they have better chances to overcome the acquired drug resistance which is a common limitation of many of the current chemotherapeutic agents.

## 4. Materials and methods

### 4.1. Chemicals, reagents and analytical methods

Chemicals, reagents and analytical grade solvents were purchased from Sigma-Aldrich (Gillingham, UK) unless specified. All reactions were carried out under argon. ^1^H and ^13^C NMR spectra were recorded in deuterated dimethyl sulfoxide (DMSO-d_6_) using a Bruker DPX 400 (400 MHz) spectrometer where chemical shifts (*δ*) were reported as parts per million (ppm) relative to tetramethylsilane (TMS) as internal standard. Coupling constants (*J*) are reported in Hertz (Hz) and multiplicities are reported as follows: s (singlet), d (doublet), t (triplet), or m (multiplet). Infrared spectra were recorded on a Perkin Elmer precisely spectrum 100 FT-IR spectrometer. Mass spectrometry data were recorded on a Thermo Fisher LTQ Orbitrap XL instrument. CHN elemental analyses for metal compounds were obtained from MEDAC LTD (Woking, UK), analytical and consultancy services.

### 4.2. Cell culture

HUVECs were purchased from Sigma-Aldrich (ECACC) (Gillingham, UK) and cultured in EGM-2 medium (EBM with SingleQuotes™ kit: foetal bovine serum (FBS), fibroblast growth factor B, epidermal growth factor, vascular endothelial growth factor (VEGF), insulin-like growth factor-1, heparin, hydrocortisone) (Lonza, Belgium). MCF-7 (ER +ve breast cancer cell line, wild type) were purchased from Sigma-Aldrich (ECACC) (Gillingham, UK) and cultured in RPMI 1640 medium supplemented with 5% FBS (Fischer Scientific, Loughborough, UK). MDA-MB-231 (triple −ve breast cancer cell line) were purchased from Sigma-Aldrich (ECACC) (Gillingham, UK) and cultured in DMEM medium (1 g L^−1^ glucose, without l-glutamine) supplemented with 2% l-glutamine (200 mM) and 10% FBS (Fischer Scientific, Loughborough, UK). The cells were incubated at 37 °C and 5% CO_2_. HUVEC cells were at passage 3–5 when used in the experiment and were not further sub-cultured. Bovine serum albumin (BSA) was purchased from Sigma Aldrich (Gillingham, UK). Recombinant human VEGFR-A165 was purchased from Peprotech (London, UK). Corning™ Matrigel™ GFR Membrane Matrix was purchased from (Fischer Scientific, Loughborough, UK). Reagents for phosphate buffered saline (PBS) for cell culture were purchased from Sigma Aldrich (Gillingham, UK). PBS (pH 7.4) was freshly prepared in lab and solution pH was checked before used. Images were captured using 1.3 M microscope digital eyepiece camera. ImageJ software was used to quantify tube formation and cell migration. The stock solutions of the test compounds (20 mM) were prepared in 100% sterile DMSO. These stocks were then appropriately diluted with the complete culture medium and DMSO levels were maintained below 0.1% in the test concentrations.

### 4.3. I-motif DNA binding study

UV-visible absorbance measurements and DNA UV melting studies were performed on a Cary 300 C (Varian USA) UV-visible spectrophotometer. VEGF and c-myc i-motif DNA (5′-3′ sequences (CCCCGCCCCCGGCCCGCCCC) and (CCTTCCCCACCCTCCCCACCCTCCCCA), respectively) were purchased from Sigma-Aldrich (Gillingham, UK) and used without further purification. Na cacodylate buffer (20 mM, pH 5.5) was used for both i-motifs. DNA concentrations were determined by measuring the absorbance at 260 nm after melting using the molar extinction coefficient supplied by the manufacturer. VEGF and c-myc i-motifs were prepared for experiments by diluting with buffer solution, annealed with or without the flavonoid at 95 °C for 5 min then gently cooled to room temperature. The stock solutions of the test compounds (20 mM) were prepared in 100% sterile DMSO. These stocks were then appropriately diluted with HPLC grade water and DMSO levels were maintained below 0.1% in the test concentrations.

### 4.4. Statistical analysis

Statistical analysis was carried out against the control group by one-way ANOVA followed by Dunnett's *post hoc* test using Graphpad Prism 6. Statistical significance value was set at *p* < 0.05.

### 4.5. Synthesis

Synthesis of the parent flavonoids (1) and (2) followed the previously published protocol.^30,33^

#### 4.5.1. Synthesis of chlorido [(5-oxo-κO)-7-hydroxy-2-(4′-chlorophenyl)-4*H*-chromen-4-onato-κO] (η^6^-*p*-cymene)ruthenium(ii) (1Ru)

A solution of NaOMe (28 mg, 0.525 mmol, 1.05 eq.) in 10 mL anhydrous MeOH was added to 2-(4-chlorophenyl)-5,7-dihydroxy-4*H*-chromen-4-one (1) (144 mg, 0.5 mmol) and the suspension was stirred at 50 °C for 30 min. [Ru(η^6^-*p*-cymene)Cl_2_]_2_ (168 mg, 0.275 mmol, 0.55 eq.) in 10 mL anhydrous DCM was added to the reaction mixture and this was stirred at 75 °C overnight under an argon atmosphere. The solvent was evaporated *in vacuo* and the residue was dissolved in 15 mL warm CHCl_3_ : MeOH (9 : 1) and filtered to remove any salts and impurities formed during the reaction. The filtrate was concentrated *in vacuo* to 2–3 mL and the product was precipitated by the addition of few drops of EtOAc. The formed precipitate was filtered, air dried and recrystallized from EtOAc : ACN (9 : 1) to give a bright red powder.

Yield: 30%; mp: decompose 230 °C; elemental analysis: found: C, 53.45; H, 4.12; Ru, 17.72. C_25_H_22_Cl_2_O_4_Ru requires C, 53.77; H, 3.97; Ru, 18.10; IR *ν*_max_/cm^−1^: 3231 (OH, w, b), 1633 (C=O, v, s), 1094 (C–O, v, s); ^1^H NMR: (400 MHz, DMSO-d_6_, Me_4_Si) *δ* 1.29 (3H, d, *J* = 2.4 Hz, CH_3_ cym), 1.30 (3H, d, *J* = 2 Hz, CH_3_ cym), 2.17 (3H, s, CH_3_ cym), 2.81–2.88 (1H, m, CH cym), 5.37 (2H, d, *J* = 7.2 Hz, H-2, 6cym), 5.66 (2H, d, *J* = 7.2 Hz, H-3, 5cym), 5.99 (1H, d, *J* = 2.4 Hz, H-6), 6.05 (1H, d, *J* = 2 Hz, H-8), 7.00 (1H, s, H-3), 7.61 (2H, d, *J* = 8.4 Hz, H-2′, 6′), 8.05 (2H, d, *J* = 8.4 Hz, H-3′, 5′), 10.27 (1H, s, OH); ^13^C NMR: (100 MHz, DMSO-d_6_, Me_4_Si) *δ* 17.92 (CH_3_ cym), 22.52 (2xCH_3_ cym), 30.89 (CH cym), 78.54 (C8), 82.86 (C6), 90.33 (C10), 97.08 (C3), 102.70 (C3, 5 cym), 106.34 (C2, 6 cym), 128.49 (C3′, 5′), 129.68 (C2′, 6′), 129.81 (C1, 4 cym), 158.58 (C2, 5), 168.09 (C7), 177.55 (C=O); *m*/*z* (FTMS + ESI): observed as M-Cl (C_25_H_22_O_4_^35^ClRu) requires 523.0250, found 523.0233.

#### 4.5.2. Synthesis of chlorido [(5-oxo-κO)-7-hydroxy-2-(4′-chlorophenyl)-4*H*-chromen-4-thionato-κO] (η^6^-*p*-cymene)ruthenium(ii) (2Ru)

A solution of NaOMe (28 mg, 0.525 mmol, 1.05 eq.) in 10 mL anhydrous MeOH was added to 2-(4-chlorophenyl)-5,7-dihydroxy-4*H*-chromene-4-thione (2) (152.37 mg, 0.5 mmol) and the suspension was stirred at 50 °C for 30 min. [Ru(η^6^-*p*-cymene)Cl_2_]_2_ (303 mg, 0.495 mmol, 0.9 eq.) in 10 mL anhydrous dichloromethane was added to the reaction mixture and stirred at 75 °C overnight under argon atmosphere. The solvent was evaporated *in vacuo* and the residue was dissolved in 15 mL warm CHCl_3_ : MeOH (9 : 1) and filtered to remove any salts and impurities formed during the reaction. The filtrate was concentrated *in vacuo* to 2–3 mL and the product was precipitated by the addition of few drops of EtOAc. The formed precipitate was filtered, air dried and recrystallized from EtOAc : CHCl_3_ (9 : 1) to give a dark reddish brown powder.

Yield: 46%; mp: >360 °C; elemental analysis: found: C, 51.58; H, 3.85; Ru, 17.30. C_25_H_22_Cl_2_O_3_RuS requires C, 52.27; H, 3.86; Ru, 17.59 (% of C content is >0.4% due to the presence of traces of chloroform); IR *ν*_max_/cm^−1^: 3141 (OH, w, b), 1173 (C=S, v, m), 1088 (C–O, v, m); ^1^H NMR: (400 MHz, DMSO-d_6_, Me_4_Si) *δ* 1.16 (3H, s, CH_3_ cym), 1.18 (3H, s, CH_3_ cym), 2.26 (3H, s, CH_3_ cym), 2.81–2.87 (1H, m, CH cym), 6.18 (1H, d, *J* = 2.4 Hz, H-6), 6.2 (1H, d, *J* = 2.4 Hz, H-8), 7.07 (2H, d, *J* = 8.4 Hz, H-2, 6 cym), 7.11 (2H, d, *J* = 8.4 Hz, H-3, 5 cym), 7.62 (1H, s, H-3), 7.64 (2H, d, *J* = 8.8 Hz, H-2′, 6′), 8.11 (2H, d, *J* = 8.8 Hz, H-3′, 5′), 10.57 (1H, s, OH); ^13^C NMR: (100 MHz, DMSO-d_6_, Me_4_Si) *δ* 21.04 (CH_3_ cym), 24.46 (2xCH_3_ cym), 33.45 (CH cym), 93.00 (C8), 111.99 (C3), 126.56 (C1′, 3′, 5′), 128.49 (C4 cym), 129.28 (C2′, 6′), 129.81 (C1 cym), 135.03 (C4′), 145.78 (C2); *m*/*z* (FTMS + ESI): observed as M-Cl (C_25_H_22_O_3_S^35^ClRu) requires 539.0022, found 539.0062.

### 4.6. Biological assays

#### 4.6.1. Cytotoxicity on HUVECs

Cytotoxic activity of treatments on HUVECs using trypan blue exclusion assay.^55^ HUVECs were seeded in 96 well plates at 1 × 10^5^ cells per mL and cultured for 24 h. Cells were then treated with either luteolin, the synthesised derivatives at 40 μM or culture medium (control) for 24 h. After 24 h, solutions were removed and cells washed with 100 μL PBS followed by the addition of 50 μL trypsin–EDTA and incubation for 5 min to detach the cells. 50 μL of EGM-2 media was added to the wells. 50 μL aliquots of the cell suspension were mixed with equal volume of trypan blue (TB) 0.2% v/v (prepared from 0.4% TB diluted with PBS) then the cells were counted using a haemocytometer. Number of viable and dead cells were counted manually and % cell viability of each treatment was expressed as % of control using the equation:% Cell Viability = (Cell viability in treatment/Cell viability in control) × 100where cell viability was calculated as follows:Cell Viability = Number of viable cells/Total number of cells(viable and dead).

#### 4.6.2. Endothelial cell tube formation assay

HUVECs were cultured until confluency and then serum starved (0.1% serum) for 24 h. The tube formation assay followed the reported assay.^56^ Briefly, 96 well plates were coated with 50 μL of Corning™ Matrigel™ GFR Membrane Matrix at 4 °C and incubated at 37 °C for 1–2 h to solidify. Serum starved HUVECs were seeded on the solidified Matrigel at 3 × 10^5^ cells per mL and treated with medium containing VEGF (10 ng mL^−1^) and either luteolin or one of the synthesised derivatives (at 1 μM or 10 μM). Plates were incubated for 12 h and photos covering the whole well area were taken using 4× magnification power of an inverted light microscope. Number of junctions, number and length of segments and master segments were quantified from the taken Images *via* the Angiogenesis Analyzer plugin^57^ in ImageJ software.^58^ The Angiogenesis Analyzer plugin proved to be an efficient tool in characterizing the branching of endothelial cells into tube networks as well as identifying various elements of endothelial tube formation.^57^ Data was represented as a ratio to the positive control (VEGF enriched).

#### 4.6.3. Cell viability

MCF-7 and MDA-MB-231 cells were seeded at a density of 4 × 10^4^ and 2 × 10^4^ cells per mL, respectively, into 96 well plates and incubated to allow the attachment for 24 h. After 24 h, the cells were treated with one of the synthesised derivatives at range of concentrations (0–100 μM) for 67 h. After 67 h, 20 μL of MTT (5 mg mL^−1^) solution in PBS was added to each well and the cells were incubated for 5 h. The purple crystals formed were dissolved in 100 μL of DMSO and the plates were read at 560 nm using a SPECTRA max UV spectrometer (Bio-Rad). The data represented are the mean of the three individual experiments.

#### 4.6.4. Scratch assay

MCF-7 or MDA-MB-231 cells were seeded in 12-well plates at 2 × 10^5^ or 1 × 10^5^ cells per mL, respectively, and cultured until 70–80% confluency. Afterwards, they were serum-starved (0% serum) for 24 h to inactivate the cell proliferation. A scratch was performed on the cell monolayers using a 200 μL pipette tip. Cells were then washed twice with PBS and treated with complete medium and one of the synthesized derivatives at 1, 10 or 20 μM. Compound 2 was not tested on MCF-7 cell line due to cytotoxicity (IC_50_ = 1.2 μM). Images of the scratches were taken immediately after performing the scratch (*t* = 0 h) and at 24 h (*t* = 24 h). The area not covered by the cells was quantified using ImageJ software. % of wound closure was calculated using the following equation:100 × ((Area of scratch at *t*0 − Area of scratch at *t*24)/Area of scratch at *t*0)

#### 4.6.5. I-motif DNA binding study

UV-visible absorbance measurements and UV melting studies of either free i-motifs (VEGF or c-myc), free flavone (1, 1Ru, 2 or 2Ru) or both i-motif DNA and flavone (2 μM: 2 μM) were recorded using 1.0 cm matched quartz cuvettes. First spectra were recorded in the range of 200–500 nm to determine the relevant wavelength range were no peaks were observed above 320 nm. Subsequent spectra were hence recorded in the range of 200–320 nm. UV-visible absorbance was measured at 25 °C while melting curves were collected at a heating rate of 1 °C min^−1^ from 20 to 90 °C. *T*_m_ was calculated by non-linear fitting of the sigmoidal curve obtained at 295 nm.

## Conflicts of interest

There are no conflicts to declare.

## Supplementary Material

MD-014-D2MD00304J-s001
