# Supplementary material for: Anticancer evaluation of new organometallic ruthenium(ii) flavone complexes
Source: RSC Med Chem. 2022 Dec 15;14(2):253–67. doi: 10.1039/d2md00304j (PMC9945865; doi:10.1039/d2md00304j)
Supplement: MD-014-D2MD00304J-s001 [file MD-014-D2MD00304J-s001.pdf]

Electronic Supplementary Material (ESI) for RSC Medicinal Chemistry.
This journal is © The Royal Society of Chemistry 2022

Anticancer evaluation of new organometallic ruthenium(II) flavone complexes

Supporting information

1. Synthesis:

1.1. Structural characterization of compounds 1 and 2 as previously reported

2-(4-Chlorophenyl)-5,7-dihydroxy-4H-chromen-4-one (1)

Yield: 97%; **mp:** 250 °C (lit.[1] 294-296 °C); **IR** $\boldsymbol{\nu_{max}}$**/cm^{-1}:** 3350 (OH, w, b), 1680 (C=O, v, m), 1091 (C-O, v, s); **^{1}H NMR:** (400 MHz, DMSO-d6, Me_4Si) δ 6.29 (1H, s, H-6), 6.59 (1H, s, H-8), 7.08 (1H, s, H-3), 7.62 (2H, d, *J* = 8.8 Hz, H-2',6'), 7.99 (2H, d, *J* = 8.8 Hz, H-3',5'), 12.20 (1H, s, OH), 12.84 (1H, s, OH); **^{13}C NMR:** (100 MHz, DMSO-d6, Me_4Si) δ 93.98 (C8), 99.04 (C6), 105.51 (C3, C10), 128.22 (C1'), 128.70 (C3', C5'), 129.18 (C2', C6'), 131.93 (C4'), 157.36 (C9), 161.39 (C5), 161.94 (C2), 166.41 (C7), 181.83 (**C**=O); ***m/z*** **(FTMS+ESI):** M+H ($C_{15}H_{10}O_4{}^{35}Cl$) requires 289.0262, found 289.0260. HPLC Purity: 99.4%

2-(4-Chlorophenyl)-5,7-dihydroxy-4H-chromene-4-thione (2)

Yield: 64%; **mp:** 247.7-248 °C (lit.[1] 249-252 °C); **IR** $\boldsymbol{\nu_{max}}$**/cm^{-1}:** 3358 (OH, w, b), 1170 (C=S, v, m), 1135 (c-O, v, m);**^{1}H NMR:** (400 MHz, DMSO-d6, Me_4Si) δ 6.28 (1H, s, H-6), 6.55 (1H, s, H-8), 7.55 (1H, s, H-3), 7.58 (2H, d, *J* = 8.4 Hz, H-2',6'), 8.11 (2H, d, *J* = 8.4 Hz, H-3',5'), 11.24 (1H, s, OH), 13.54 (1H, s, OH); **^{13}C NMR:** (100 MHz, DMSO-d6, Me_4Si) δ 95.28 (C8), 101.30 (C6), 113.13 (C10), 118.00 (C3), 129.84 (C1', 3', 5'), 129.84 (C2', C6'), 137.63 (C4'), 153.62 (C2), 154.55 (C9), 162.32 (C7), 165.19 (C5), 196.52 (**C**=S); ***m/z*** **(FTMS+ESI):** M+H ($C_{15}H_{10}O_3{}^{35}ClS$) requires 305.0034, found 305.0034. HPLC Purity: 98.6%

1.2. NMR spectra

(A)

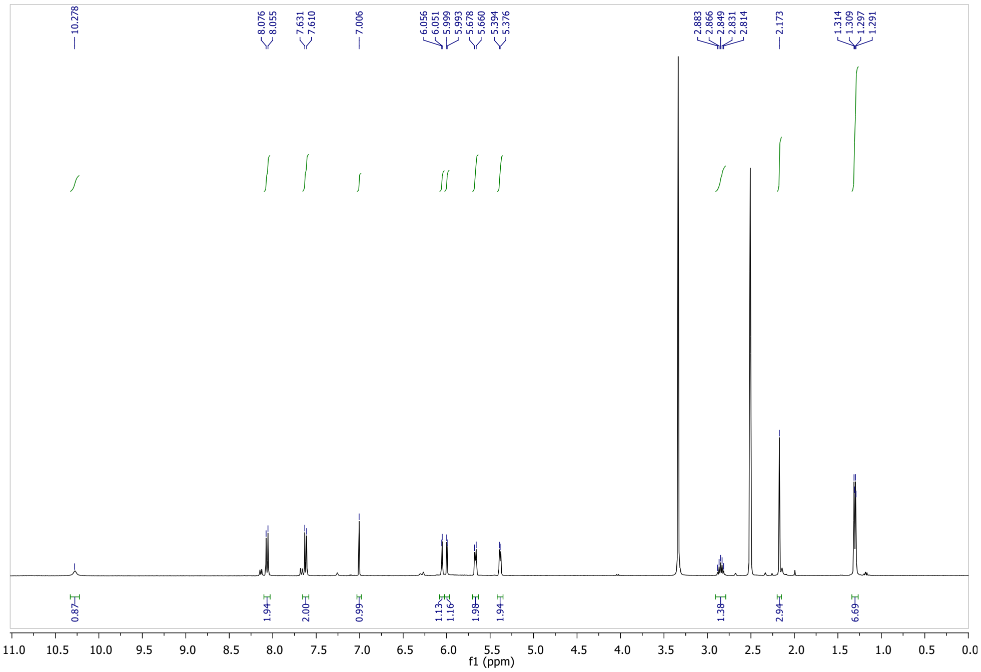
10.278
8.076
8.055
7.631
7.610
7.006
6.056
6.051
5.999
5.993
5.678
5.660
5.394
5.376
2.883
2.866
2.849
2.831
2.814
2.173
1.314
1.309
1.297
1.291
0.87
1.94
2.00
0.99
1.13
1.16
1.98
1.94
1.38
2.94
6.69
f1 (ppm)

(B)

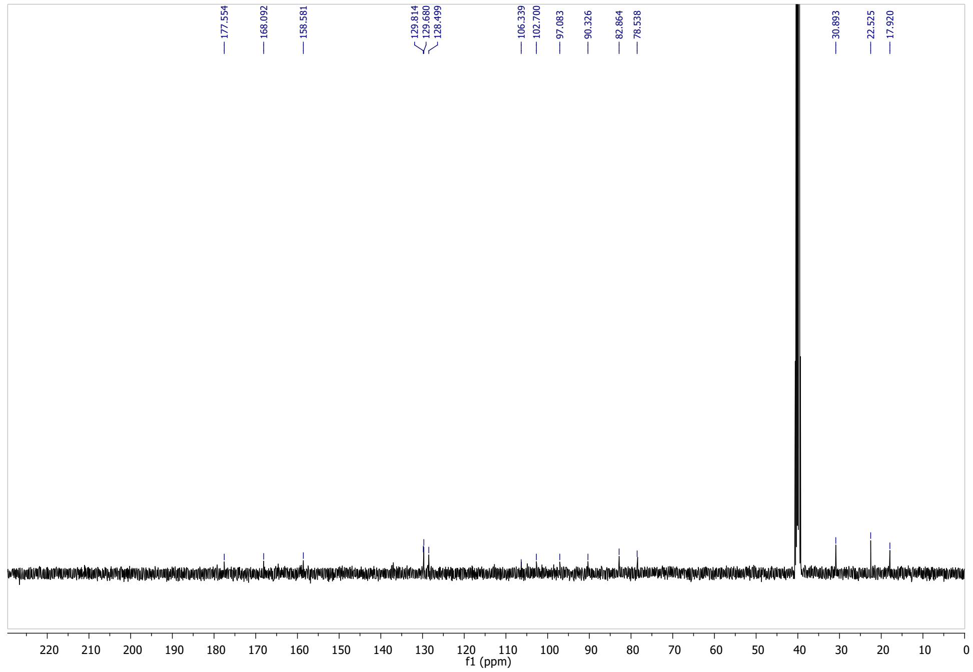
177.554
168.092
158.581
129.814
129.680
128.499
106.339
102.700
97.083
90.326
82.864
78.538
30.893
22.525
17.920
f1 (ppm)

(C)

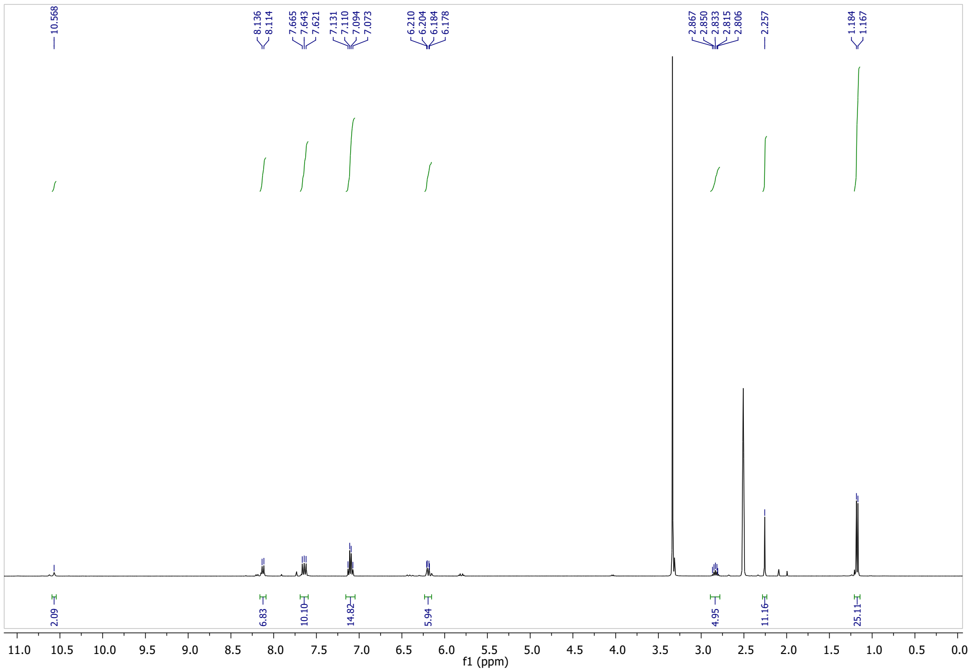

(D)

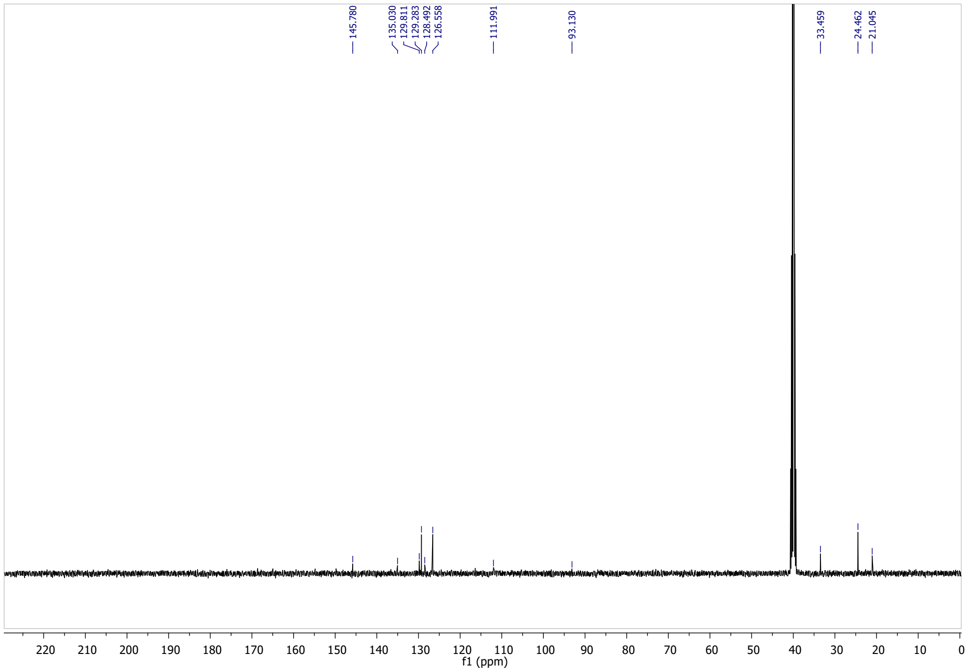

Figure 1. ^{1}H NMR spectra of Ru complexes (1Ru and 2Ru). (A) 1Ru ^{1}H NMR; (B) 1Ru ^{13}C NMR; (C) 2Ru ^{1}H NMR; (D) 2Ru ^{13}C NMR.

(A)

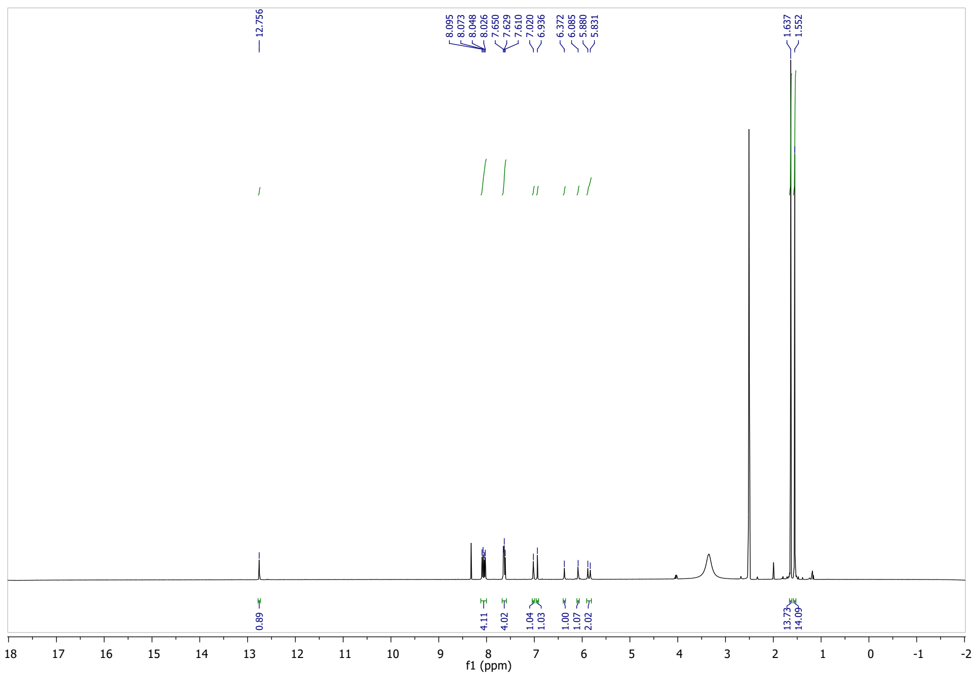

(B)

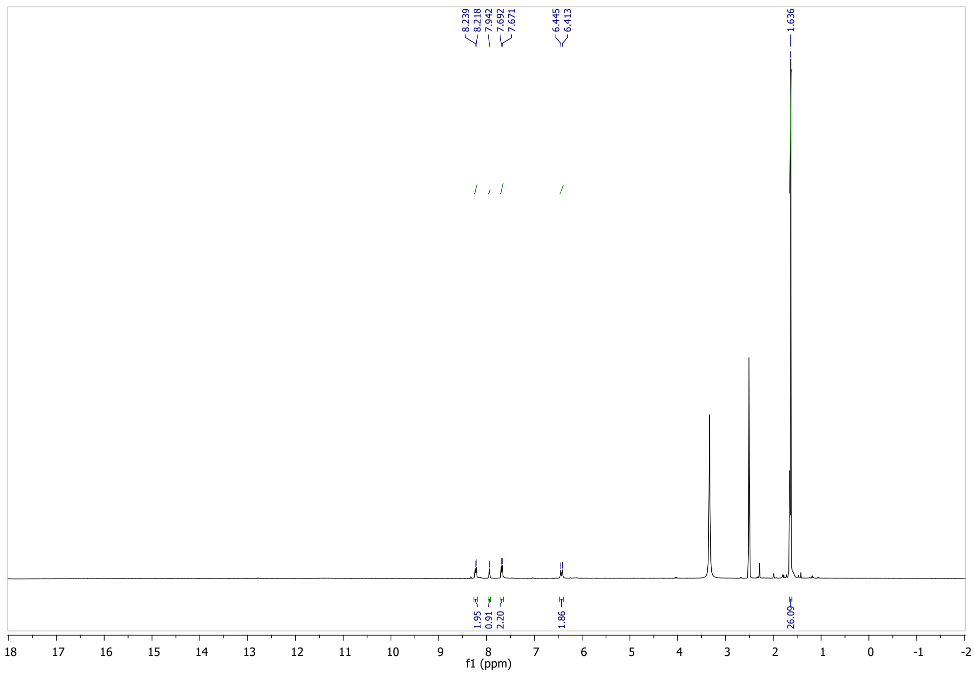

Figure 2. ^{1}H NMR spectra of (A) 1Ir and (B) 2Ir.

2. UV-VIS stability profiles

(A) (B)

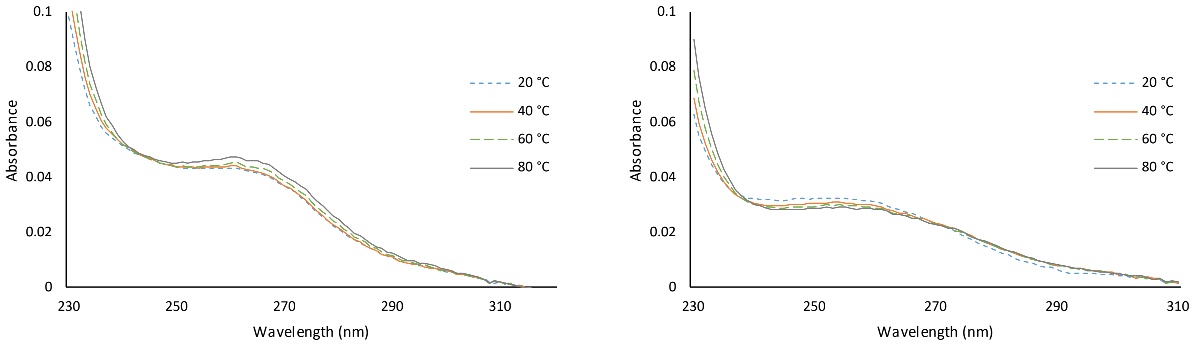

Figure 3. Temperature dependant UV-VIS spectra of (A) 1Ru and (B) 2Ru in 0.1%DMSO/ddH_2O solution recorded over a 6 h time interval.

Table 1. λ_{260}[a] and $\Delta\ \lambda_{260}$[b] of complexes 1Ru and 2Ru at 20 °C – 80 °C

**1Ru**	**λ_{260}**	**$\Delta\ \lambda_{260}$**	**2Ru**	**λ_{260}**	**$\Delta\ \lambda_{260}$**
**20 °C**	0.0435	0	**20 °C**	0.03108	0
**40 °C**	0.04422	0.00072	**40 °C**	0.02973	0.0013
**60 °C**	0.04525	0.00174	**60 °C**	0.02902	0.0021
**80 °C**	0.04738	0.00388	**80 °C**	0.02832	0.0028

a λ_{260} is the absorbance at 260 nm
b $\Delta\ \lambda_{260} = \lambda_{260}$ (40, 60 or 80 °C) - λ_{260} (20 °C)

References:

1. Ravishankar, D.; Watson, K. A.; Greco, F.; Osborn, H. M. I. Novel Synthesised Flavone Derivatives Provide Significant Insight into the Structural Features Required for Enhanced Anti-Proliferative Activity. *RSC Adv.* **2016**, *6* (69), 64544–64556. https://doi.org/10.1039/c6ra11041j.
